# High Tunable BaTi_x_Zr_1-x_O_3_ Films on Dielectric Substrate for Microwave Applications

**DOI:** 10.3390/molecules27186086

**Published:** 2022-09-18

**Authors:** Andrei Tumarkin, Evgeny Sapego, Alexander Gagarin, Artem Karamov

**Affiliations:** Department of Physical Electronics and Technology, Saint Petersburg Electrotechnical University “LETI”, 197022 Saint Petersburg, Russia

**Keywords:** barium titanate–zirconate, rf magnetron sputtering, initial stages of growth, microwave properties

## Abstract

In this study, the structural and microwave properties of BaTiZrO_3_ films deposited on alumina substrate were investigated. The films were deposited by RF magnetron sputtering in Ar/O_2_ ambient atmosphere. The research of the island films at the initial stages of the growth showed that the pyramidal type of growth prevails. It was demonstrated that as-deposited film is a BaZrTiO_3_ solid solution with a deficiency of titanium compared to the target. The air annealing at temperatures of 1100–1200 °C leads to the formation of a well-formed crystalline solid solution of BaZr_0.3_Ti_0.7_O_3_ with a predominant orientation (*h*00). The investigation of microwave parameters of the films fabricated at different conditions showed that the best performance with the tunability of 4.6 (78%), and the Q-factor of 18 to 40 at 2 GHz was achieved at annealing temperature of 1150 °C.

## 1. Introduction

Ferroelectrics (FE) are materials with a strong dependence of the dielectric permittivity on the applied electric field that makes them promising for microwave applications. Electrically tunable capacitors, phase shifters, and delay lines can be implemented on the basis of ferroelectric materials [1,2,3,4,5]. In comparison with semiconductors, the advantages of FE are high operating power and low losses at microwaves [6,7]. The most studied ferroelectric material for microwave applications is a solid solution based on barium and strontium titanates Ba_x_Sr_1-x_TiO_3_ (BST) [1,2].

Despite the widespread implementation, BST thin films have the inherent problem of high dielectric loss that can be partly attributed to the unstable oxidation state of titanium that can be easily reduced from Ti^4+^ to Ti^3+^ [8,9] and crucially restricts the practical applications [10,11]. There is another barium–titanate-based ferroelectric solid solution where the titanium atom in the perovskite unit cell may be replaced by a zirconium atom—BaTi_x_Zr_1-x_O_3_ (BZT). Unlike BST, where both components of the solid solution (BaTiO_3_ and SrTiO_3_) are nonlinear dielectrics, BZT is mixed from ferroelectric BaTiO_3_ and linear dielectric BaZrO_3_. BaTi_x_Zr_1-x_O_3_ obtained by substituting Ti ion at the B site of BaTiO_3_ with Zr one in compounds of the perovskite structure ABO_3_ is a possible alternative to BST. Ti^4+^ is substituted by chemically more stable Zr^4+^ [12,13,14,15], which not only suppresses the conduction by electron hopping between Ti^4+^ and Ti^3+^, but also reduces dielectric loss [16,17,18,19]. Moreover, the BZT system is known to change significantly with Zr content and exhibits a pinched phase transition at x about 0.2, i.e., all the three phase transitions that correspond to pure BaTiO_3_ are merged together or pinched into one broad peak [13,14,15,20,21,22]. BZT composition with x = 0.2 also demonstrates very good dielectric nonlinearity at room temperature [13,14,15]. BZT is potentially promising for microwave electrically tunable applications also, but has been little studied from this point of view.

A number of papers have been published, in which the structural and electrophysical properties of solid solutions with titanium replacement by zirconium are investigated both in bulk and in thin film form [12,16,18,21,23,24,25,26,27,28,29,30,31,32,33,34,35,36]. Barium titanate–zirconate films were obtained by laser evaporation [23,24,25,26,27,28,29,30,31,32], ion-plasma sputtering [16,18,33,34], and sol-gel technology [21]. In the vast majority of works, experimental data on dielectric properties in BZT structures are given for the frequency range of 1 kHz–1 MHz.

It should be noted that the high dielectric nonlinearity of capacitive elements based on BZT films is demonstrated for sandwich “metal-dielectric-metal” (MDM) structures, i.e., in cases when the film is formed on a conductive electrode [18,21,23,26,27,28,29,31,32]. The capacitance of an MDM capacitor, where the gap value is determined by the thickness of the film (usually 100–500 nm), is easily controlled by low bias voltages, but precisely because of this, the use of MDM tunable capacitors is possible in small-signal devices only. One of the main potential advantages of FE devices over semiconductor analogues, namely the ability to operate at high power levels as an electrically tuned element, can only be realized in a planar design on a dielectric substrate. In the works devoted to the studies of BZT planar ferroelectric structures on dielectric substrates, when both electrodes are formed on the FE layer, data on tunability of the order of 50% are given [16,24,25,30,33,34]. The best result for today on the tunability of planar BZT capacitive elements at 75% (at 1 kHz) was published in [34] on a rather expensive single crystal MgO substrate, which is not an optimal substrate for microwave applications due to cost, low mechanical strength, and hygroscopy. In addition, the same authors [33] provide data on significant degradation of tunability and losses of BZT/MgO planar capacitors at microwaves. Therefore, additional researches are needed to obtain improved tunability of BZT thin films on dielectric substrates with high Q-factor and low cost.

As one of such substrate material, perspective for microwave applications, polycrystalline aluminum oxide can be proposed. Alumina has excellent mechanical and dielectric properties: high mechanical hardness and chemical stability, thermal coefficient of linear expansion 8 × 10^−6^ K^−1^, which is close to BZT one, high thermal conductivity coefficient of about 30 W/m·K, stable dielectric permittivity 9.7, low losses at microwaves (tan δ < 10^−4^ at 10 GHz), and extremely low cost [4]. Today, there is no information about tunable BZT planar elements on alumina substrate in the literature.

In this regard, the purpose of this work is to search for technological approaches that allow to obtain thin layers of barium titanate–zirconate that exhibit high nonlinearity on alumina substrate, to study the structure and dielectric properties of thin BZT layers, with a view to their further application as part of high-power microwave nonlinear elements.

## 2. Experiment

The study of the structural properties of BZT films on dielectric substrates consisted of two stages: the study of the nucleation stage of the film formation on the substrate in order to understand the mechanisms of its growth, and the study of the structural and electrical properties of solid films and capacitors based on them. Deposition of the films was carried out by RF magnetron sputtering of a ceramic target of the composition BaTi_0.8_Zr_0.2_O_3_ on aluminum oxide substrates. Before the deposition process, the target was pre-sputtered away from the substrate holder for 30 min in order to clean the surface. The temperature of the substrate T_s_ during deposition was controlled using a thermocouple located under the substrate holder and varied in the range of 700—880 °C for different series of samples. An Ar/O_2_ mixture was used as the working gas, the oxygen content in the mixture varied from 25 to 100%. The working gas pressure was ranged from 2 to 10 Pa.

At the stage of investigation of the initial stages of film growth, the formation time of island structures was 60 s at a working gas pressure of 10 Pa. The temperature of the substrates varied in the range of 700–880 °C. After deposition, the samples were cooled in the atmosphere of the working gas at atmospheric pressure at a rate of 2–3 °C/min.

The structure of island films was studied by the medium energy ion scattering (MEIS) method, which is a modification of the widely used Rutherford ion backscattering (RBS) method and differs from the latter in the range of ion energies of the probing beam (units—tens of MeV in the RBS and tens-hundreds of keV in the MEIS). The reduction of the ion beam energy makes it possible to obtain a high, up to 0.5 nm, depth resolution, which is especially important when studying the initial stages of film growth [37].

In this work, He^+^ ion beams were used to study the films, and an electrostatic analyzer was used to register them. The scattering angle was 120°. Energy spectra of scattered ions were obtained for each sample in the mode of random orientation of the beam Y_r_(E). Based on the Y_r_(E) dependence, by comparing experimentally measured and calculated spectra, the composition of films, their thickness, as well as the degree of film coating of the substrate were determined.

Solid BZT films were deposited on substrates heated to 800 °C. The deposition was started at a working gas pressure of 10 Pa, which was reduced until 2 Pa for the first 30 min of deposition. The total time of deposition was 3 h, and the thickness of films was 500 nm. After deposition, the films were annealed in a tubular furnace at various temperatures in the air for 2 h. The temperature and time of heating, annealing and cooling of the samples were controlled by the temperature regulation and control system TRM-251 (OVEN-PROM, St. Petersburg, Russia).

The crystal structure and phase composition of solid films were studied by X-ray diffraction (XRD) using a DRON-6 diffractometer (Burevestnik, St. Petersburg, Russia) on the emission spectral line Cu Kα1 (λ = 1.5406 Å). Powder diffraction method in Bragg–Brentano geometry was used. The scanning was performed from 20 to 60 degrees with 0.2-degree step.

For electrical studies, planar capacitors with a gap width of 5 µm were formed on the basis of BZT films. The upper electrodes of the capacitors were made by thermal deposition of a 1 µm Cu film with an adhesive chromium sublayer, followed by lithography and chemical etching. Measurements of capacitance C and quality factor Q = 1/tan δ were performed at a frequency 2 GHz using a half-wave strip resonator and HP 8719C vector analyzer. The resonator provides an unloaded Q-factor of 1000 (the accuracy of measuring capacitance and Q-factor is 1 and 5%, respectively), as well as the possibility of supplying a control voltage up to 1000 V. The tunability of the capacitors was calculated as the ratio of capacitances at zero and maximum applied control voltage (*n* = C(0 V)/C (U_max_)) and additionally as *n* = (C_max_ − C_min_)/C_max_ to compare the results with other works.

## 3. Results and Discussion

### 3.1. Initial Stages of BZT Film Growth

The source of important information, which is necessary to understand the mechanisms of growth of solid films, is the study of the initial stages of their formation. It is well known that the properties of thin films (orientation and dimensions of crystallites, stoichiometry of the composition of the film and the presence of inclusions of secondary phases) significantly depend on the conditions under which the nucleation of the film on the substrate is carried out [38]. At the initial stage of film growth, depending on the density of the flow of atoms reaching the substrate, as well as depending on the temperature, structure and composition of the substrate, the lifetime of adatoms on surface can vary widely, determining the mechanisms of mass transfer, nucleation and, consequently, the formation of films [37]. Thus, at low substrate temperatures, as a rule, the mechanism of surface diffusion of adatoms prevails, and at high temperatures the mechanism of diffusion in the gaseous phase prevails. If a surface diffusion mechanism is implemented on the substrate, a time-constant source of atoms will lead to the lateral growth of islands (their area will grow faster than the height). For the case when the diffusion mechanism through the gaseous phase is realized in the system, the islands will mainly grow in height, forming a columnar structure [38].

When studying the initial stages of the growth of barium titanate–zirconate films, the variable technological parameters for the formed samples were the substrate temperature and the ratio of argon and oxygen in the working gas. Data on the relative content of components, the height of islands, the degree of substrate covered by film, the total amount of material on the substrate are given in Table 1. Here, h_cov_ (h on covered segments) is the thickness of the film on the coated areas of the substrate; C is the degree of the substrate covered by film; Amount is an estimate of the total amount of the deposited material BaTiZrO_3_ in units of 10^15^ united atoms per cm^2^, where the united atom is a molecule of Ba_a_Ti_b_Zr_c_O_d_, where (a + b + c + d) = 1. The height of the peaks of Ba, Zr, and Ti relative to the signal level of the substrate is determined by the degree of coverage, the width of the peaks is determined by the average thickness on the covered areas, and the ratio of amplitudes is determined by the elemental composition. The elemental composition (Ba, Ti, and Zr content) in island films determined by the MEIS method corresponds to the composition of the sputtered target with an accuracy of 5%.

**Figure 1:**
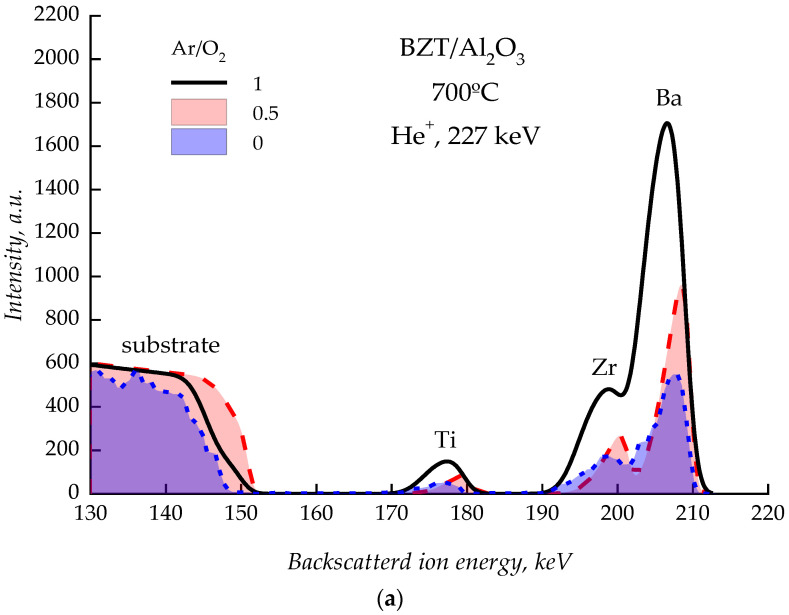

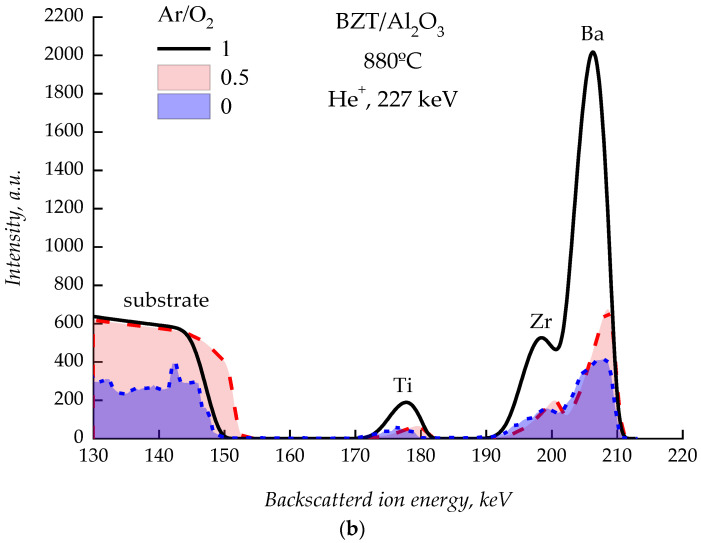
Spectra of backscattered He^+^ ions from BZT island films deposited at different compositions of the working gas at substrate temperatures of 700 °C (**a**) and 880 °C (**b**).

The energy spectra of backscattered He^+^ ions from BZT island films deposited in various gas mixtures at substrate temperatures of 700 and 880 °C are shown in Figure 1. The spectra have no significant differences, which indicates a single mechanism of nucleation in the studied ranges of gas compositions and temperatures. The triangular shape of peaks with a tightened low-energy front in the spectrum of backscattered ions indicates the pyramidal form of BZT islands on the substrate surface [37].

**Figure 2:**
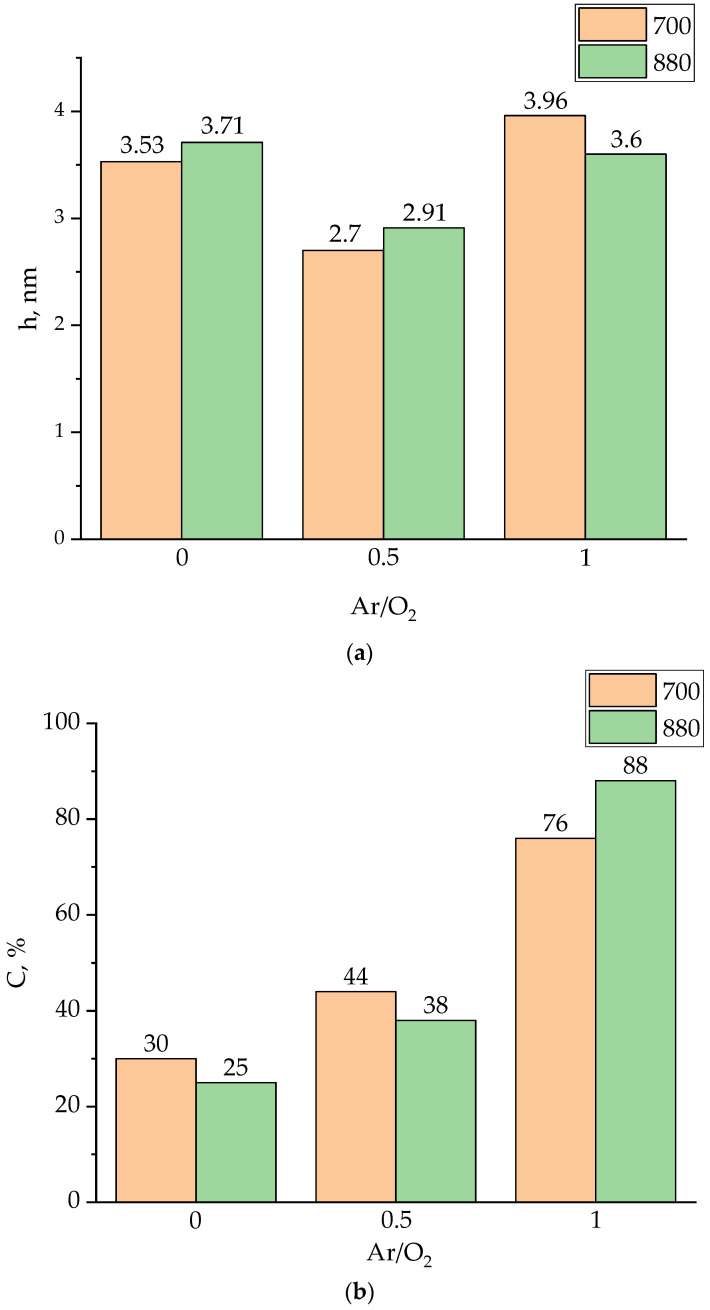
Dependence of the height of the islands h (**a**) and the area of substrate covered by film C (**b**) on the composition of the working gas at substrate temperatures of 700 °C and 880 °C.

Let us analyze the dependence of the thickness of BZT island films h (island heights) and the substrate area covered by islands C on the composition of the working gas for films deposited at substrate temperatures of 700 and 880 °C. These parameters can give an information about the mechanisms of mass transfer of adatoms before they join to an island during the initial stage of film growth. For example, an increase in the average height of the islands with a simultaneous reduction in the area of the substrate occupied by them indicates a change in the mechanism of film growth from “layer-by-layer” to “island” one [39]. In our case, the analysis indicates that an increase in the concentration of argon in the composition of the working gas does not lead to a change in the heights of the islands on the film-covered areas of the substrate surface (Figure 2a), while the area of substrate surface covered by film increases (Figure 2b).

Since the heights of the islands are quite large and do not change significantly when the deposition temperature changes, it can be concluded that the mechanism of mass transfer of adatoms through the gaseous phase prevails under these conditions. This mechanism ensures the pyramidal type of growth of BZT films [40]. An increase in the substrate area covered by film with raising in the argon content in the working gas is due to the more intensive sputtering of the target by heavier argon atoms. An increase in the sputtering rate leads to an increase in the density of the flow of atoms reaching the substrate, which, in turn, determines a more intensive filling of the substrate with islands.

Thus, the studied temperature range of deposition of barium titanate–zirconate films can be considered as a range in which the mass transfer of adatoms through the gaseous phase prevails, which determines the formation of a columnar film structure at the initial stage of the growth. The presence of such a structure on the substrate suggests that with further growth of the film at temperatures considered, a predominantly oriented phase may form [38,39]. A change in the ratio of argon and oxygen in the composition of the working gas does not change the shape of the islands, but affects the deposition rate of the film.

### 3.2. Structure Characterization of BZT Films

**Figure 3:**
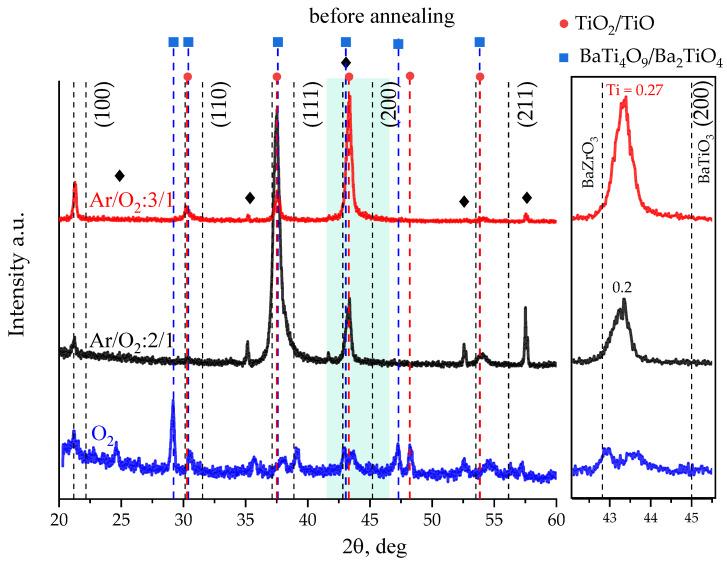
Diffractograms of BZT thin films on Al_2_O_3_ substrates deposited at different oxygen concentrations in gas ambient at a substrate temperature of 800 °C.

Figure 3 shows diffractograms of solid BZT films deposited in various gas mixtures at a substrate temperature of 800 °C. Dotted lines on the left indicate the positions of reflexes for pure BaZrO_3_ and on the right for BaTiO_3_. The reflexes from the substrate are marked with diamonds. A number of factors attract attention to themselves: (1) For film deposited in pure oxygen medium the polycrystalline highly defective layer consisted of barium zirconate (22, 31, and 43 deg) and probable impurity phases of titanium oxides (PDF 8–117, 21–1272, and 29–1360 marked by red dotted lines) and barium polytitanates, presumably Ba_2_TiO_4_ and BaTi_4_O_9_ (PDF 35–813 and 34–70 marked by blue dotted lines), is formed. (2) For films deposited at a reduced oxygen content in the working gas, a shift of the angular positions of the reflexes towards large angles is observed, which indicates the formation of BaZrTiO_3_ solid solution with a deficiency of titanium compared to the target. In addition, a decrease in the oxygen content in the gas mixture leads to a significant increase in the intensity of BZT reflexes, which means an improvement in the crystal structure of the solid solution. A decrease in the oxygen concentration in the working gas does not exclude the possibility of the presence of titanium oxides and barium polytitanates secondary phases on the substrate along with the main phase. (3) Films deposited in a gas mixture Ar/O_2_ 2:1 and Ar/O_2_ 3:1 differ in BZT texture: (111) and (100), respectively.

According to Figure 3, sputtering of the BaTi_0.8_Zr_0.2_O_3_ ceramic target in an oxygen-containing gas mixture does not lead to the formation of the same BZT solid solution on the substrate. A similar situation is described in [33], where sputtering of the BaTi_0.7_Zr_0.3_O_3_ target in a 9:1 Ar/O_2_ gas mixture and at similar deposition temperatures led to the formation of a BZT film with a lattice constant of 4.12 Å, which corresponds to a solid solution of BaTi_0.3_Zr_0.7_O_3_. The authors make an assumption about the presence of oxygen vacancies in the film, which increase the lattice parameter of it in comparison with the target. In our case, two facts prevent the confirmation of this supposition. (1) If oxygen vacancies are the reason for the increase in the lattice parameter of the deposited film, then deposition in an environment with a high concentration of oxygen should lead to a decrease in their concentration and, accordingly, to a decrease in the lattice parameter of the formed layer. However, it does not happen. (2) The MEIS elemental analysis confirms the almost stoichiometric transfer of the target components to the substrate (see Table 1). In other words, the system on the substrate does not experience a shortage of elements for the formation of a solid solution. Then, the probable explanation for the discrepancy between the composition of the solid solution of the film and the target is the presence of secondary phases on the substrate along with BZT.

Thus, according to XRD data, as well as [21,33,41,42], the formation of solid solutions with the substitution of the position of titanium in the perovskite cell on dielectric substrates in an oxygen-containing medium is difficult, which is apparently due to the presence of secondary titanium and barium oxides on the substrate. The rate of the formation of these oxides exceeds the rate of the formation of zirconium ones (redox potentials of barium, titanium and zirconium in this case are −2.9 V, −1.75 V, and −1.53 V, respectively) [43]. A decrease in the oxygen content in the working gas leads to the suppression of the formation of secondary titanium-containing oxides and to an increase in the concentration of titanium in the BZT solid solution.

**Figure 4:**
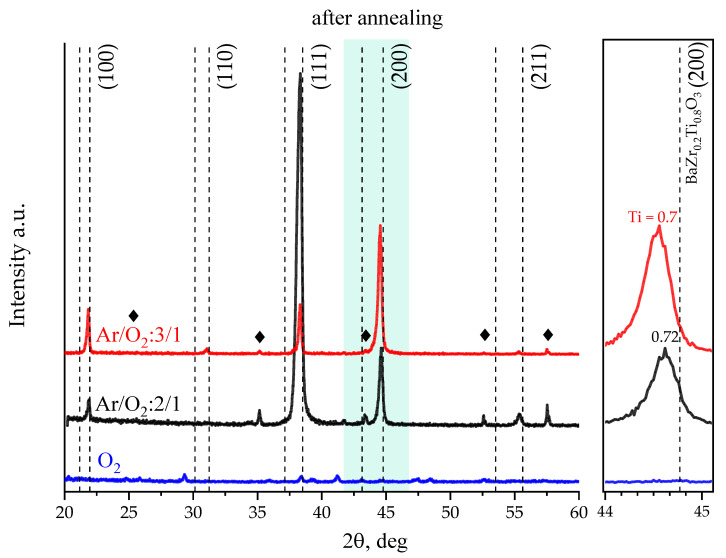
Diffractograms of BZT thin films on Al_2_O_3_ substrates deposited at different oxygen concentrations in gas ambient at a substrate temperature of 800 °C after annealing at 1100 °C.

Figure 4 shows diffractograms of the films described above, but subjected to high-temperature annealing in air at a temperature of 1100 °C for two hours (here dotted lines on the right indicate the positions of reflexes for BaTi_0.8_Zr_0.2_O_3_—the composition of the sputtered target). The data of X-ray diffraction analysis indicate a shift of the angular positions of reflexes towards large angles as a result of annealing. The reflex shift shows a decrease in the unit cell parameter from 4.11 Å to 4.07 Å due to the introduction of Ti^+4^ ions with a small ionic radius into the solid solution lattice.

Thus, post-growth high-temperature treatment radically changes the structural properties of the studied films. Since the temperature of formation of a solid solution of BZT is significantly higher than the temperature of formation of both BaTiO_3_, BaZrO_3_, and simple titanium oxides [35,43], then during high-temperature annealing, titanium is redistributed between the secondary phases of titanium-containing oxides and BZT to form a solid solution of the composition BaZr_0.3_Ti_0.7_O_3_ (see inset with reflex (200)). In addition, for films deposited in a gas medium with a reduced oxygen content, annealing leads to a significant increase in the intensity of dominant reflexes with a simultaneous decrease in their integral width, which indicates an improvement in the quality of the crystal lattice of the coatings under study.

**Figure 5:**
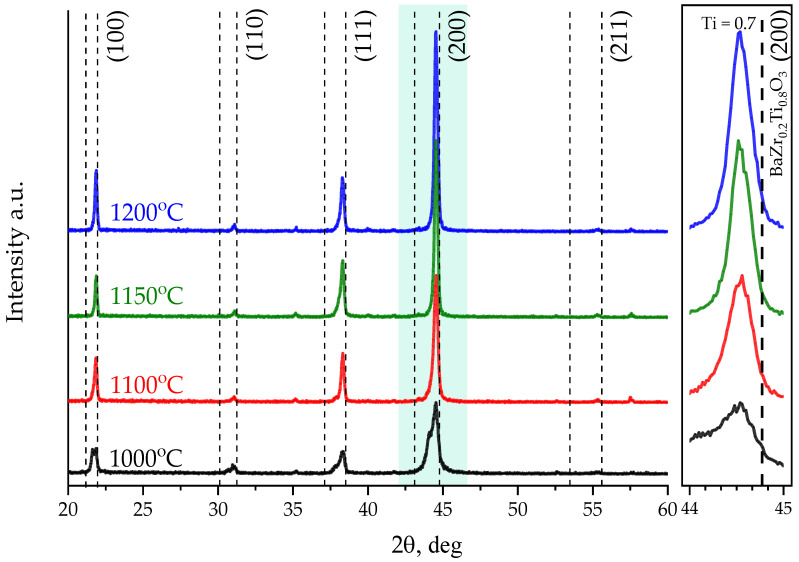
Diffractograms of BZT thin films on Al_2_O_3_ substrates deposited in the gas mixture Ar/O_2_:3/1 after annealing at different temperatures.

Figure 5 shows comparative diffractograms of BZT films deposited in an Ar/O_2_:3/1 gas mixture and subjected to high-temperature annealing at various temperatures. According to XRD analysis, films annealed at temperatures of 1100–1200 °C are a well-formed crystalline solid solution of BaZr_0.3_Ti_0.7_O_3_ with a predominant orientation (*h*00) without signs of inclusions of secondary phases.

A diffractogram of a sample annealed at a temperature of 1000 °C looks different. Attention is drawn to relatively weak peak intensities, tightened leading edges, as well as doublets on 23 and 44 degrees, which indicates the presence of secondary phases of barium polytitanates and crystal lattice defects in the film.

### 3.3. Electrical Properties

**Figure 6:**
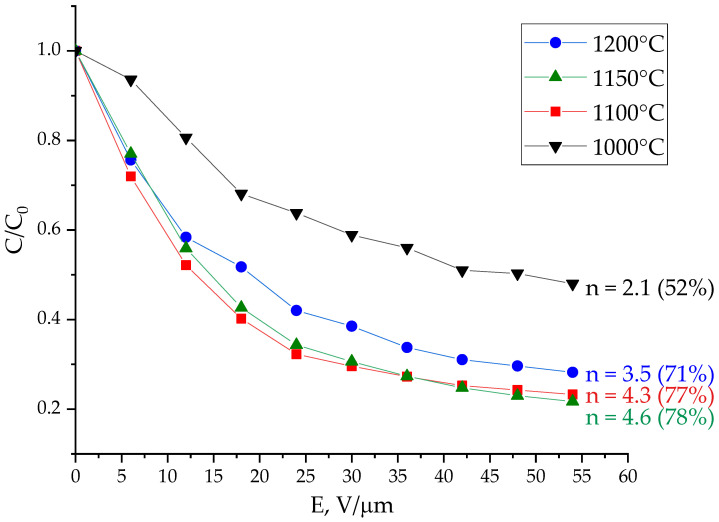
Voltage–capacitance characteristics of planar capacitors based on BZT films subjected to annealing at various temperatures.

Planar capacitors were formed on the basis of BZT films deposited in the gas mixture Ar/O_2_:3/1 and annealed at various temperatures. The dependences of the capacitance normalized to the maximum value on the strength of the control field of the capacitors under study are shown in Figure 6. It follows from the graph that the capacitor based on the film annealed at 1000 °C exhibits the least tunability, which is explained by the defects in the crystal lattice of the solid solution and the possible presence of secondary phases (see Figure 5). The tunability of capacitors formed on the basis of films annealed at temperatures of 1100, 1150, and 1200 °C is 4.3, 4.6, and 3.5, respectively. The decrease in the nonlinearity of capacitor based on the film subjected to annealing at maximum temperature is apparently due to the occurrence of mechanical defects on the surface of the films (cracks) as a result of annealing, which is explained by the difference in the temperature expansion coefficients of the BZT film and the substrate. The tunability of the capacitor 4.6 times (78%) is the best result for planar BZT capacitors today.

**Figure 7:**
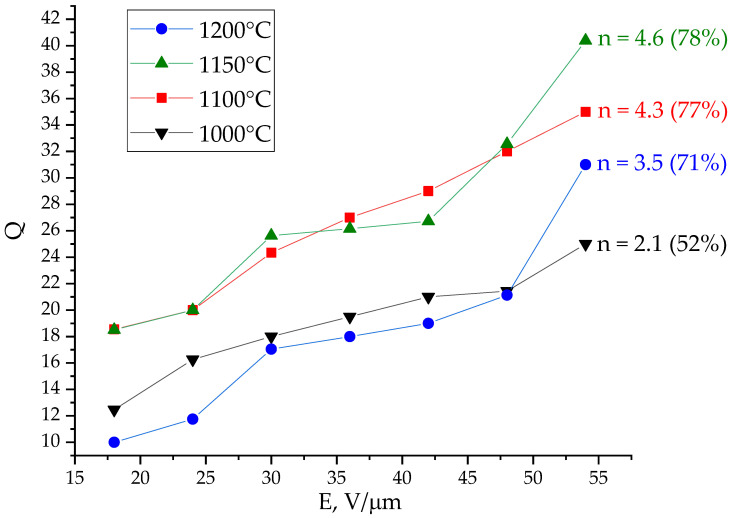
The 2 GHz Q-factor of planar capacitors based on BZT films subjected to annealing at various temperatures.

The dependences of the Q-factor of the studied capacitive structures on the strength of the control field measured at a frequency of 2 GHz are shown in Figure 7. Two groups of curves can be distinguished on the graph. (1) The Q-factor of capacitors based on films annealed at temperatures of 1100 and 1150 °C increases from 18 to 40 under the action of an applied field, which can be explained by a well-formed predominantly oriented crystal structure of a solid solution (see Figure 5). (2) The relatively low Q-factor of capacitors based on films formed by annealing at 1000 and 1200 °C is due to the defective polycrystalline structure of BZT films in the first case, when grain boundaries make an additional contribution to dielectric losses, and mechanical defects in films resulting from annealing at 1200 °C.

Table 2 presents comparative data on the tunability of MDM and planar capacitors based on barium titanate-zirconate films. It follows from the table data that the capacitors obtained in this work exhibit high dielectric nonlinearity at microwaves, which is the best result published today for both sandwich and planar capacitive structures based on BZT films. Taking into account the fact that the high tunable BZT films were grown for the first time on alumina substrates, this result looks promising for microwave electrically tunable applications.

## 4. Conclusions

The study of the initial stages of BZT films growth has shown that in the temperature range of 700–880 °C the pyramidal type of growth prevails. An increase the argon content in the composition of the working gas does not change the mechanism of film growth, but increases the rate of film deposition.

It was demonstrated that the BZT film as-deposited at a reduced oxygen content in the working gas on Al_2_O_3_ substrate is a BaZrTiO_3_ solid solution with a deficiency of titanium compared to the target. High-temperature annealing of films investigated leads to the formation of a crystalline solid solution of BaZr_0.3_Ti_0.7_O_3_ with a structure and electrical properties depended on the annealing temperature. BZT films annealed at 1100–1150 °C have a well-formed crystal lattice with a predominant orientation (*h*00) at the absence of secondary phase inclusions; their component composition is close to the composition of the target, which has a positive effect on their electrophysical properties, in particular on the nonlinearity and the level of dielectric losses.

The tunability of capacitors formed on the basis of films annealed at a temperature of 1150 °C of 4.6 times (78%) is the best result for planar BZT capacitors today. A comparison of the results obtained with the literature data showed that planar BZT structures on alumina substrate exhibit promising characteristics for an elaboration of high-power microwave tunable devices.

## Figures and Tables

**Table 1:** Characteristics of island films investigated.

No.	Ar/O_2_	T_s_	Ba	Zr	Ti	O	h_cov_, nm	C, %	Amount
2264	0	880	0.198	0.039	0.163	0.60	3.71	25	7.5
2265	0.5	880	0.199	0.040	0.161	0.60	2.91	38	7.3
2266	1	880	0.199	0.041	0.160	0.60	3.6	88	7.6
2267	0	700	0.199	0.039	0.162	0.60	3.53	30	7.4
2268	0.5	700	0.199	0.040	0.161	0.60	2.7	44	7.2
2269	1	700	0.200	0.040	0.160	0.60	3.96	76	7.5

**Table 2:** Data on the tunability of MDM and planar capacitors based on barium titanate–zirconate films.

Composition	Substrate	Construction	Tunability, %	Reference
Mn-BaTi_0.8_Zr_0.2_O_3_	Pt/Si	MDM	69	[26]
BaTi_0.65_Zr_0.35_O_3_	Pt/Si	MDM	42	[21]
BaTi_0.8_Zr_0.2_O_3_	Pt/Si	MDM	69	[32]
BaTi_0.85_Zr_0.15_O_3_	CaRuO_3_/Pt/Si	MDM	75	[28]
BaTi_0.8_Zr_0.2_O_3_	LaSrMnO_3_/Pt/Si	MDM	73	[29]
BaTi_0.8_Zr_0.2_O_3_	Pt/Si	MDM	70	[23]
BaTi_0.8_Zr_0.2_O_3_	Sn doped In_2_O_3_	MDM	53	[27]
BaTi_0.8_Zr_0.2_O_3_	F doped SnO_2_	MDM	44	[27]
BaTi_0.8_Zr_0.2_O_3_	Pt/Si	MDM	59	[27]
BaTi_0.8_Zr_0.2_O_3_	Pt/LaAlO_3_	MDM	44	[18]
BaTi_0.9_Zr_0.1_O_3_	Pt/LaAlO_3_	MDM	59	[18]
BaTi_0.8_Zr_0.2_O_3_	LaAlO_3_/Sr_2_AlTaO_6_	planar	50	[24]
BaTi_0.8_Zr_0.2_O_3_	MgO	planar	50	[25]
Mn-BaTi_0.8_Zr_0.2_O_3_	MgO	planar	53	[30]
BaTi_0.7_Zr_0.3_O_3_	MgO	planar	76 (1 MHz)	[34]
**BaTi_0.7_Zr_0.3_O_3_**	**Al_2_O_3_**	**planar**	**78** (**3 GHz**)	**This work**

## Data Availability

Not applicable.
